# TUFT1 is expressed in breast cancer and involved in cancer cell proliferation and survival

**DOI:** 10.18632/oncotarget.20472

**Published:** 2017-08-24

**Authors:** Weiguang Liu, Lei Zhang, Zining Jin, Min Zhao, Zhan Li, Guanglei Chen, Lisha Sun, Bo Chen

**Affiliations:** ^1^ Department of Breast Surgery, The First Hospital of China Medical University, Shenyang 110001, Liaoning Province, China; ^2^ Department of Breast Surgery, The Second Hospital of Dalian Medical University, Dalian 116000, Liaoning Province, China

**Keywords:** TUFT1, breast cancer, proliferation, apoptosis, prognosis

## Abstract

Tuftelin 1 (TUFT1), which plays an important role in the initial stages of the mineralization of ectodermal enamel, is widely expressed in different embryonic and adult tissues and some tumor cells. However, since the roles of this gene have not been thoroughly investigated in tumors, its function in the development of breast cancer remains unclear. We proved both human specimens studies and cell line studies, that TUFT1 expression levels are increased in breast cancer samples, and the increased expression of TUFT1 was shown to be positively correlated with tumor size, histological grade, lymph node metastasis rate, and poor prognosis. Further *in vitro* studies showed that the inhibition of TUFT1 expression in T-47D and MDA-MB-231 breast cancer cells significantly affected cell proliferation, induced apoptosis, and led to G1-phase cell cycle arrest. Moreover, reduced TUFT1 expression restrained tumor growth compared with the control group *in vivo*. Furthermore, microarray and pathway analysis demonstrated that TUFT1 inhibition led to significant changes of several signaling pathways and semi-quantitative western blot analysis showed that a decrease in TUFT1 expression was accompanied by changes in MAPK signaling pathway components. The obtained results suggest that TUFT1 may represent a novel breast cancer marker and a potentially effective therapeutic target.

## INTRODUCTION

Breast cancer is one of the most common cancers and it represents the main cause of cancer-related death in women [1]. Breast cancer is considered a heterogeneous disease, not only based on its clinical and pathological properties, but based on the differences in the molecular features as well [2]. Various mechanisms and different bioactive compounds are involved in the induction of breast carcinogenesis and metastasis. Signal transduction pathways can be affected by these molecules, which can further lead to the changes in gene expressions, resulting in abnormal cell proliferation, differentiation, and growth. Ultimately, this may induce carcinogenesis. In order to elucidate molecular mechanisms underlying the development of breast cancer, it is very important to identify a potent and effective diagnostic marker. The understanding of these molecular mechanisms is expected to lead to an improved treatment of breast cancer.

Tuftelin 1 (TUFT1) was originally identified as a molecule involved in the growth and maturation of extracellular enamel, leading to the mineralization of the epithelial tissue of the teeth in vertebrates [3]. TUFT1 is expressed in the morula, embryonic stem cells, soft tissues, such as brain (especially in neurons), kidneys, adrenal gland, liver, testis, and tumor cells. It was reported that TUFT1 may be perform certain functions in the mesenchymal stem cells, and to be involved in the differentiation of neural cells, which is mediated by nerve growth factor [4, 5]. Furthermore, a previous study found that 1% O_2_ treatment of human HepG2 and MCF-7 cell lines induced TUFT1 expression in the hypoxic environment, which was shown to be necessary for tumorigenesis [6]. The results obtained in these studies demonstrate that TUFT1 is likely to have various functions in different tissues, and the alterations in these normal functions can promote the development of certain diseases and tumors. However, the role of TUFT1 in tumors has not been investigated. Our analysis of differentially expressed genes in cancer and adjacent tissue samples, using the Cancer Genome Atlas (TCGA) database, containing the information about a large number of clinical breast cancer samples, showed that TUFT1 expression is significantly increased in many breast cancer samples. Therefore, the aim of this study was to investigate the alterations in the expression and biological functions of TUFT1, and correlate these findings with breast cancer patient prognosis.

## RESULTS

### TUFT1 expression is increased in breast cancer tissue samples and it correlates with poor prognosis

**Figure 1:**
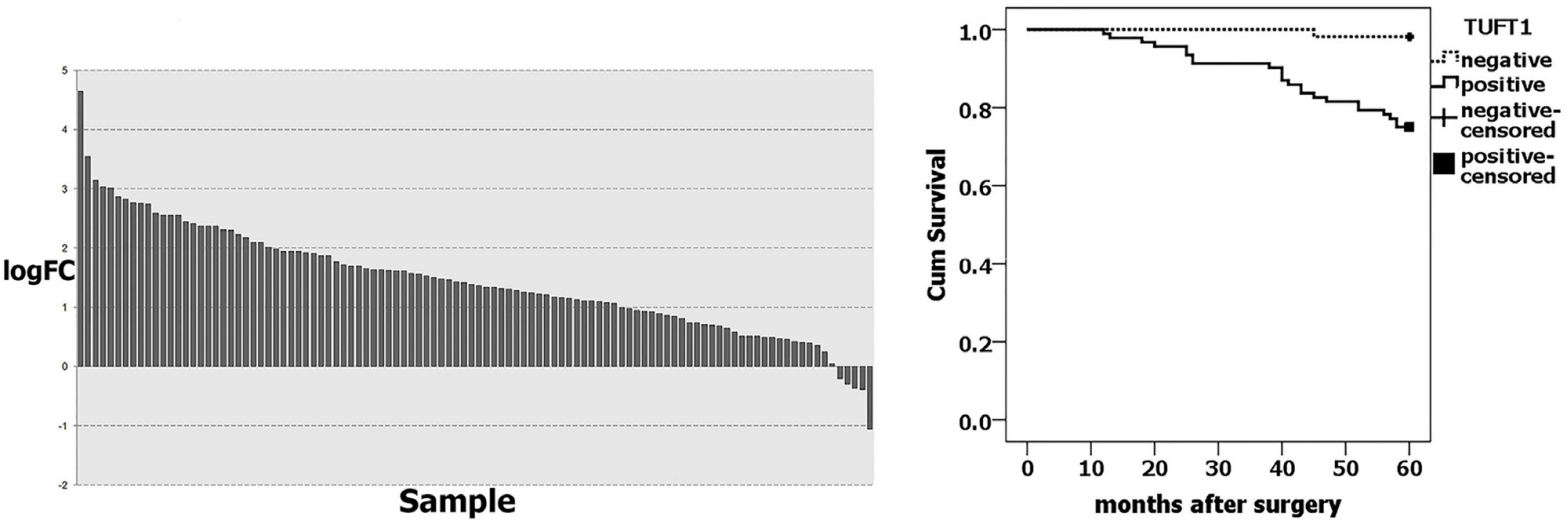
TUFT1 expression was correlated with prognosis of breast cancer **(A)** TUFT1 expression in TCGA Breast (P=7.77× 10^-55^, n=106). logFC = Log_2_ Cancer/Normal, **(B)** TUFT1 was associated with breast cancer-specific survival in 146 cases (No loss of follow-up, P = 0.000, log-rank test).

We analyzed TUFT1 expression in large datasets from The Cancer Genome Atlas (TCGA) databases. The TCGA RNA Seq data showed that TUFT1 was significantly upregulated in over 67.92% of breast cancer tissues (*n* = 106) compared with adjacent normal breast tissues (P=7.77E-55) (Figure 1A). Immunohistochemistry (IHC) analyses showed that TUFT1 positivity rate was 63.0% (92/146) in the cancer tissue samples, which was significantly higher than that observed in the adjacent normal breast tissues (16.7%; 10/60 samples)(P= 0.000, Table 1). The increased expression of TUFT1 was shown to be positively correlated with tumor size, histological grade, lymph node metastasis (P = 0.010, P =0.000, P =0.000, respectively; Table 1). Our patient follow-up analysis showed that, in total, 24 out of 146 patients died, and the 5-year survival rate was 83.6%. Out of these 24 patients, in 23, TUFT1 was shown to be expressed, while only one person died in the negative-expression group. Kaplan-Meier analysis (Figure 1B) revealed that the survival rate of patients with TUFT1 expression was significantly lower than the survival of patients shown not to express TUFT1 in malignant tissue (P= 0.000).

**Table 1:** The relationship between TUFT1 expression and the clinicopathological factors (n =146)

Varible	n	TUFT1^-^	TUFT1^+^	P varible
**Tissue**				0.000
**Cancer tissue**	146	54	92	
**Adjacent tissue**	60	50	10	
**Age**				0.176
**≥40**	125	49	76	
**<40**	21	5	16	
**Tumor size**				0.010
**T1**	56	26	30	
**T2**	78	28	50	
**T3, 4**	12	0	12	
**Histological grades**				0.000
**I**	31	16	15	
**II**	77	34	43	
**III**	38	4	34	
**Lymph node metastasis**				0.000
**Negative**	64	35	29	
**Positive**	82	19	63	

**Figure 2:**
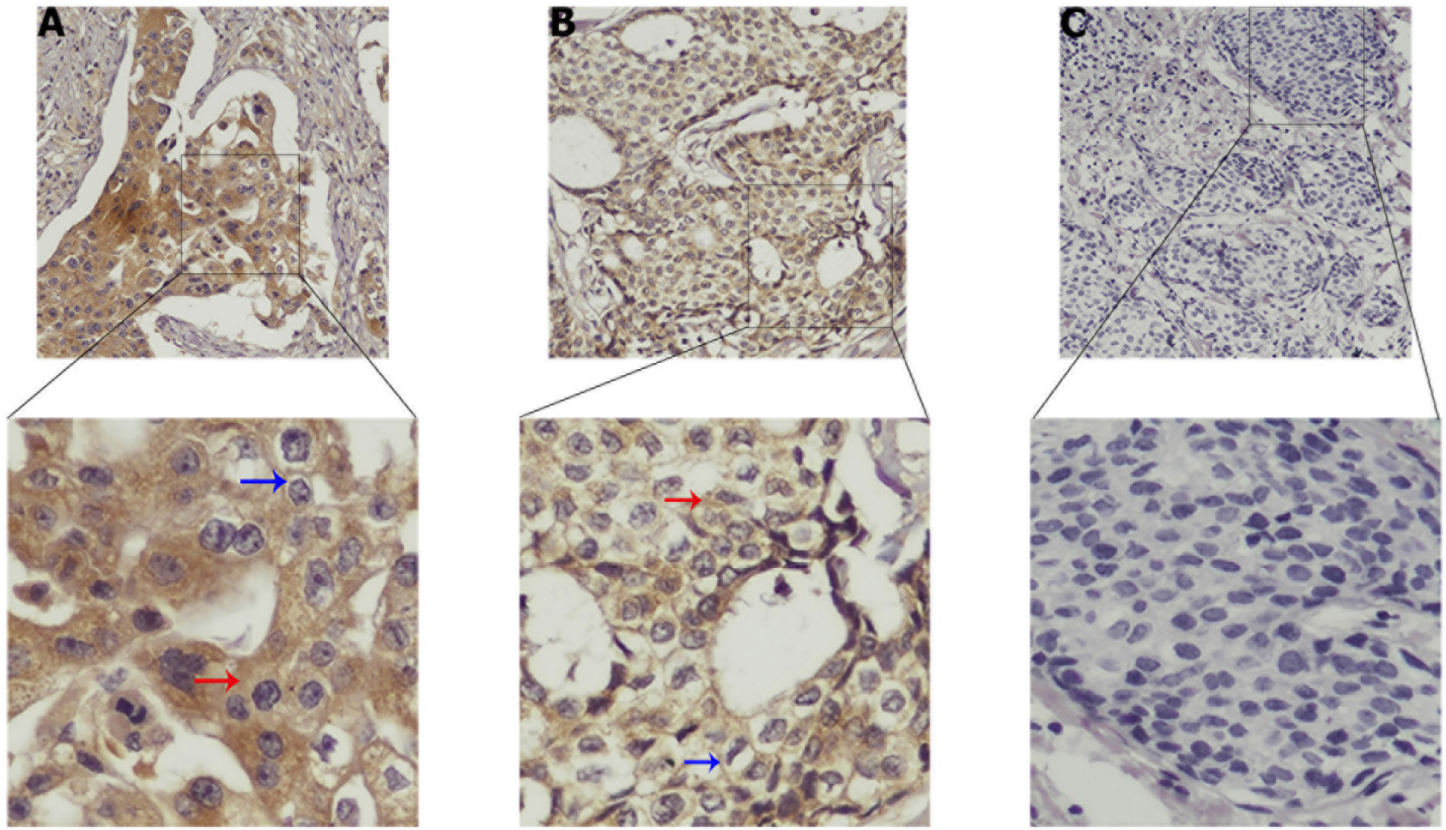
Strong, medium and negative expressions of TUFT1 are shown in **(A–C)**, respectively. Blue arrow represents TUFT1 staining in the cytomembrane, red arrow represents TUFT1 staining in the cytoplasm**.**

TUFT1 was shown to be expressed mainly in cytoplasm and cytomembrane of breast cancer cells. As shown in Figure 2A-2C, TUFT1 expression was shown to be strong, weak, or negative, respectively.

### TUFT1 knockdown in breast cancer cells affects their proliferation, and induces apoptosis and cell

**Figure 3:**
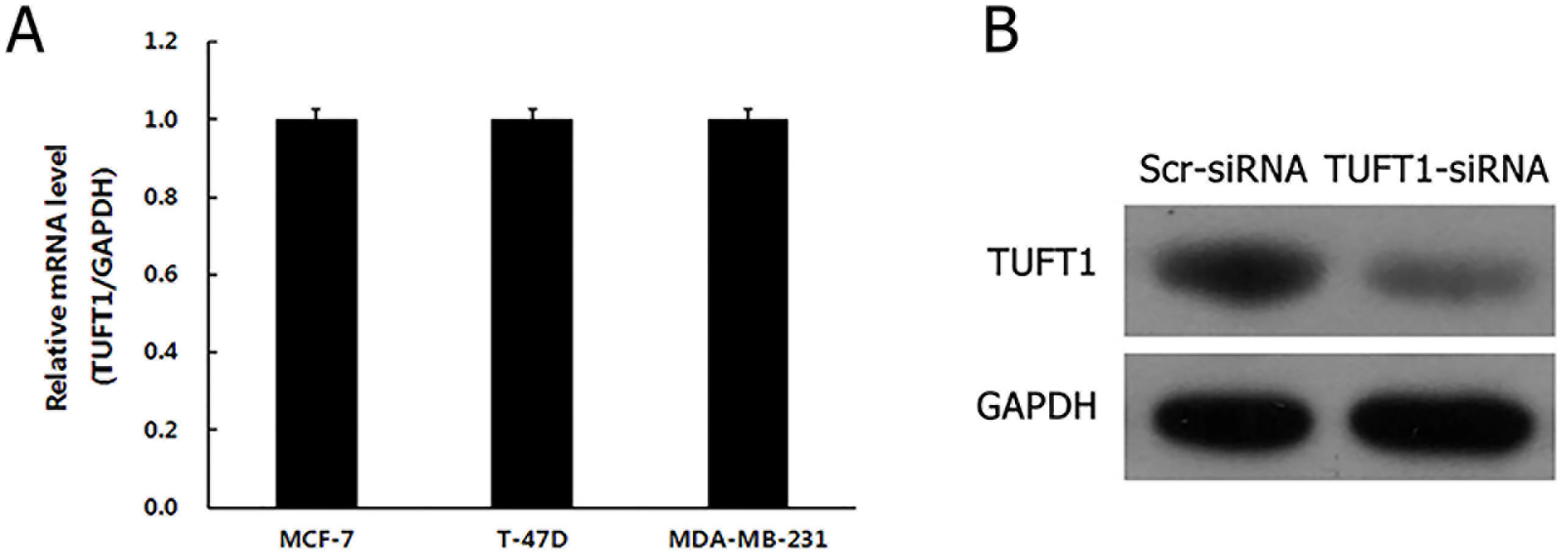
**(A)** Levels of TUFT1 mRNA were analyzed by Real time-PCR in three breast cancer cell lines. **(B)** Protein levels of TUFT1 in 293T cells measured by western blot.

We analyzed the expression levels of TUFT1 (Figure 3A) in MCF-7, T-47D, and MDA-MB-231 cells, and showed that TUFT1 is expressed here. T-47D and MDA-MB-231cell lines were selected for subsequent knockdown studies.

**Figure 4:**
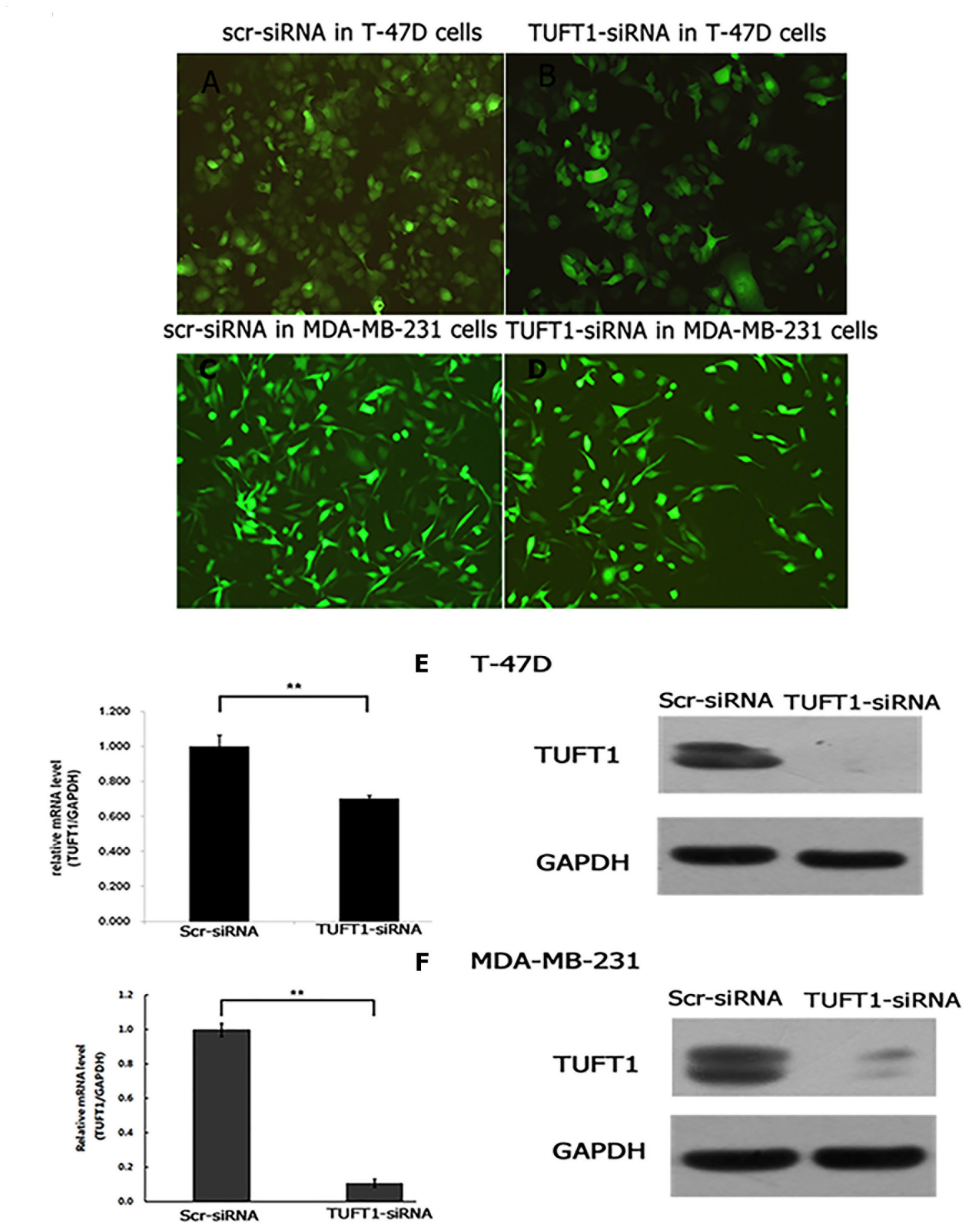
T-47D and MDA-MB-231 cells infected with TUFT1-siRNA or scr-siRNA lentivirus were examined by fluorescent microscopy microscopy at the 3rd day after infection **(A-D).** **(E)** Both of mRNA and protein level of TUFT1 knockdown were detected respectively by real-time quantitative PCR and western blot in T-47D cells based on lentiviral-based siRNA. **(F)** Both of mRNA and protein level of TUFT1 knockdown were detected respectively by real-time quantitative PCR and western blot in MDA-MB-231cells based on lentiviral-based siRNA. Data shown are the mean ±S.D. (**t test P<0.01 as compared to control groups, n = 3, respectively).

Initially, TUFT1 expression was inhibited by the infection of 293T cells with TUFT1-small interfering RNA (siRNA)-carrying lentiviral particles (TUFT1-knockdown group), or scrambled (scr)-siRNA-carrying lentiviruses, as a control. Western blot analyses showed that TUFT1 expression in TUFT1-knockdown cells was significantly decreased in comparison with that in the control cells (Figure 3B). Afterward, we performed TUFT1 knockdown in T-47D and MDA-MB-231 cells, and showed that the efficiency of the knockdown, for both TUFT1-siRNA and scr-siRNA was greater than 80% at 72 h after the infection (Figure4A-4D). TUFT1 mRNA and protein levels were evaluated by using real-time PCR and western blot, which showed that these levels were significantly decrease in TUFT1-knockdown cells, in comparison with those in the control cells (Figure 4E, 4F).

**Figure 5:**
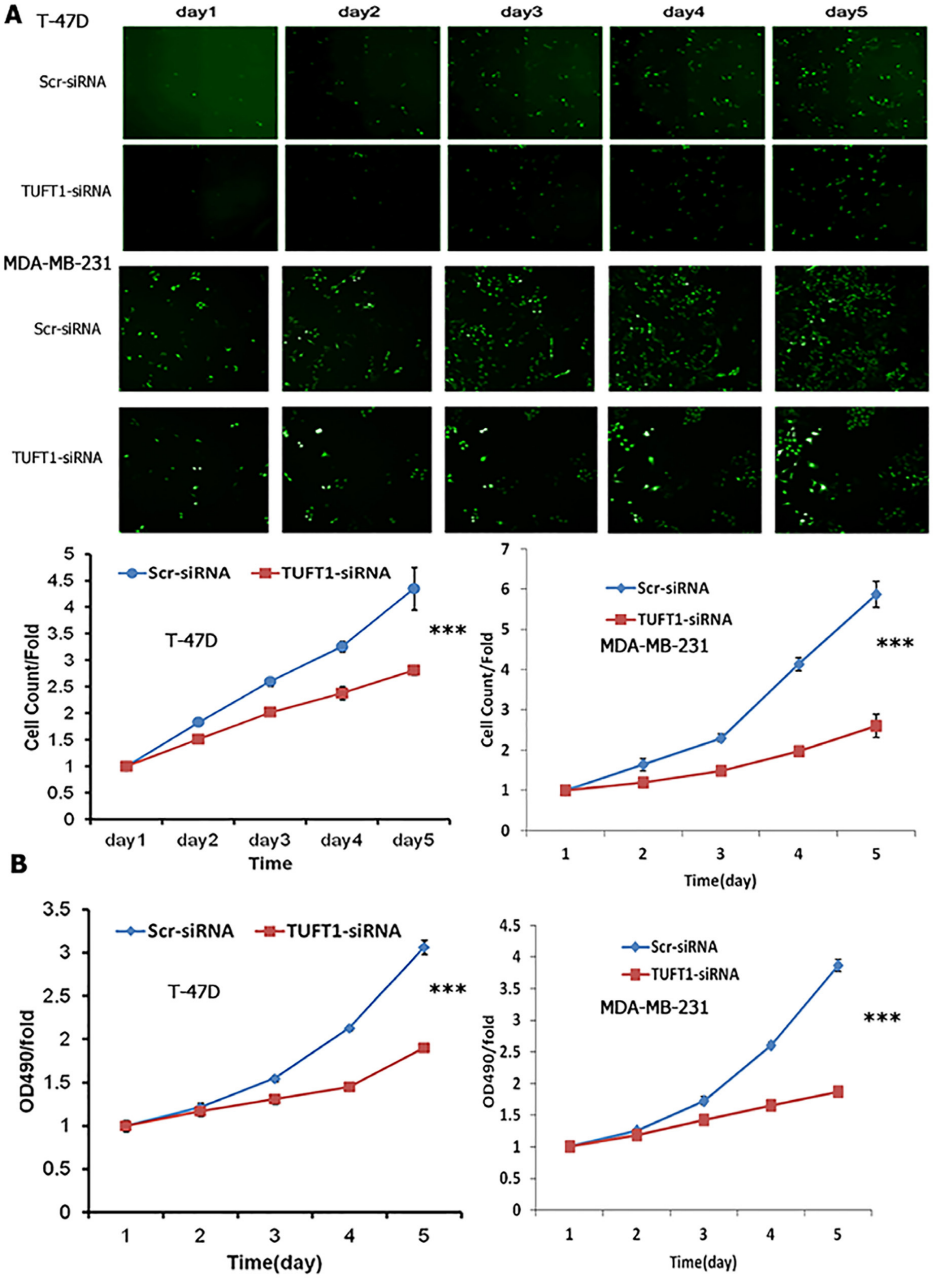
Cell proliferation with TUFT1 knockdown in T-47D and MDA-MB-231 cells were detected by Cellomics Array Scan VT1 and MTT assay **(A)** High content cell imaging assays were applied to acquire raw images of cell growth rate. **(B)** Cell growth rate was monitored by MTT assay on the 2nd, 3rd, 4th and 5th days in two type of cells. Data shown are the mean ±S.D. (***t test P <0.001, n = 3, respectively).

The proliferation rates of T-47D and MDA-MB-231 cells, infected with either TUFT1-siRNA or scr-siRNA, were analyzed using high-content screening (HCS) and MTT assay. Downregulation of TUFT1 was shown to induce a reduction in cell number during 5 days of analysis, and significantly decrease cell proliferation rate (P<0.001, Figure 5A). Similar results were obtained in MTT assay (P<0.001, Figure 5B).

**Figure 6:**
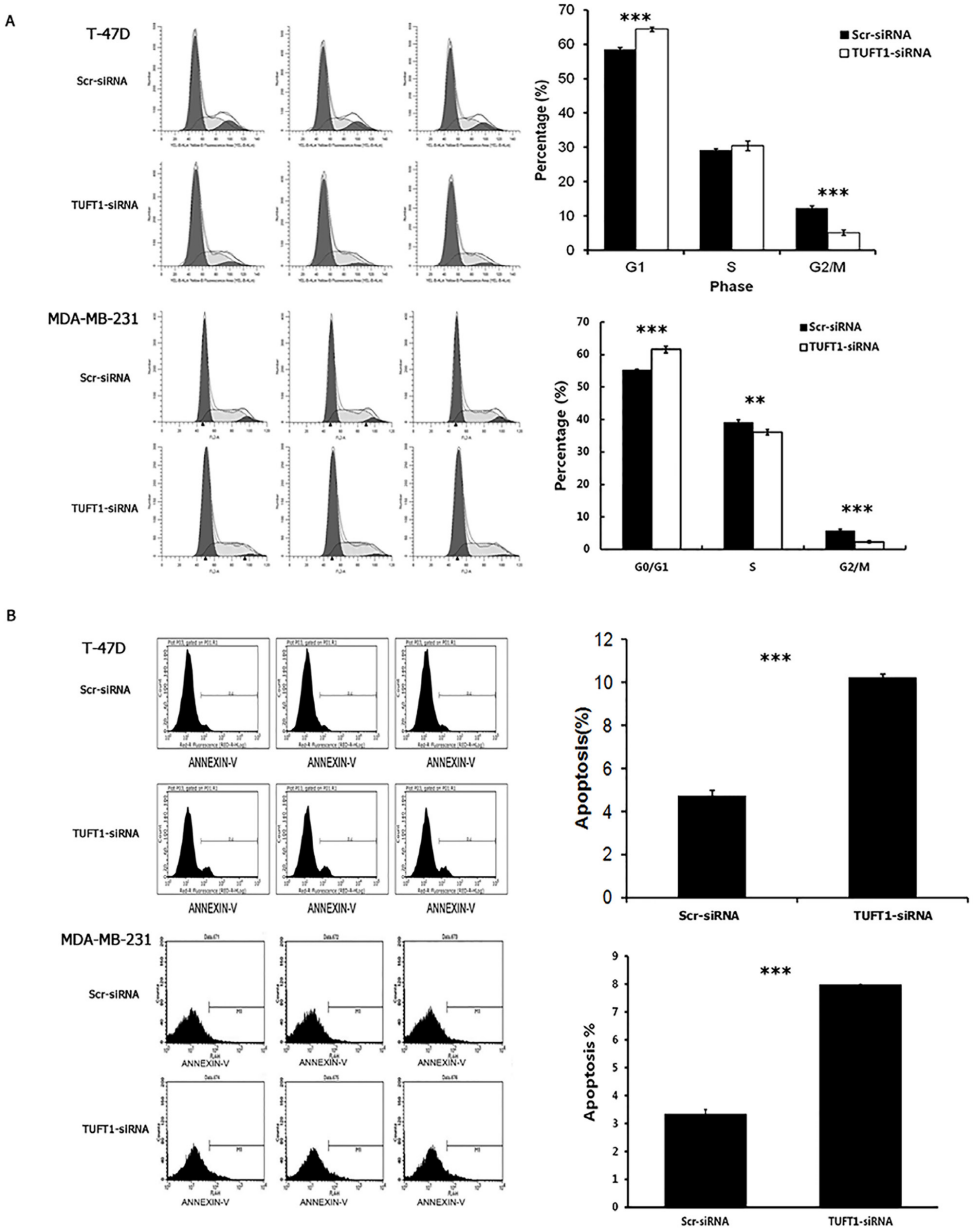
Knockdown of TUFT1 expression induced G1phase arrest and cell apoptosis in T-47D and MDA-MB 231 cells **(A)** Cell cycle of T-47D and MDA-MB 231cells was analyzed and G1 cell cycle phase was determined by flow cytometry. **(B)** Quantification of cell apoptosis in cells infected with lentivirus expressing Scr-siRNA or TUFT1-siRNA. Data shown are the mean ±S.D. (**t test P <0.01, *** P <0.001, n = 3, respectively).

The role of TUFT1 in T-47D and MDA-MB-231 cell cycle progression was determined by analyzing the distribution of these cells across the cell cycle phases, using flow cytometry. As shown in Figure 6A, compared with that in the control group, the percentage of T-47D and MDA-MB-231 cells in G0/G1 phase in the TUFT1-kncokdown group was significantly increased (58.59 ± 0.48% *vs*. 64.44 ± 0.63%, 55.26 ± 0.25% *vs*. 61.55 ± 1.03%, respectively; P <0.001), and adverse results were obtained in G2/M phase (12.25 ± 0.71% *vs*. 5.15 ± 0.83%, 5.69 ± 0.58% *vs*. 2.35 ± 0.34%, respectively; P < 0.001).

The rate of the apoptosis of TUFT1-kncokdown T-47D and MDA-MB-231cells was analyzed using AnnexinV staining. We demonstrated that, in comparison with that in the control group, apoptosis was significantly induced in the TUFT1-knockdown T-47D group (4.72 ± 0.23% *vs*. 10.25 ± 0.15%, respectively; P <0.001). Similarly, the rate of apoptosis was increased in TUFT1-knockdown MDA-MB-231cells (3.34 ± 0.17% *vs*. 7.97 ± 0.02%, respectively; P <0.001) (Figure 6B).

### TUFT1 knockdown inhibits tumor growth *in vivo*

**Figure 7:**
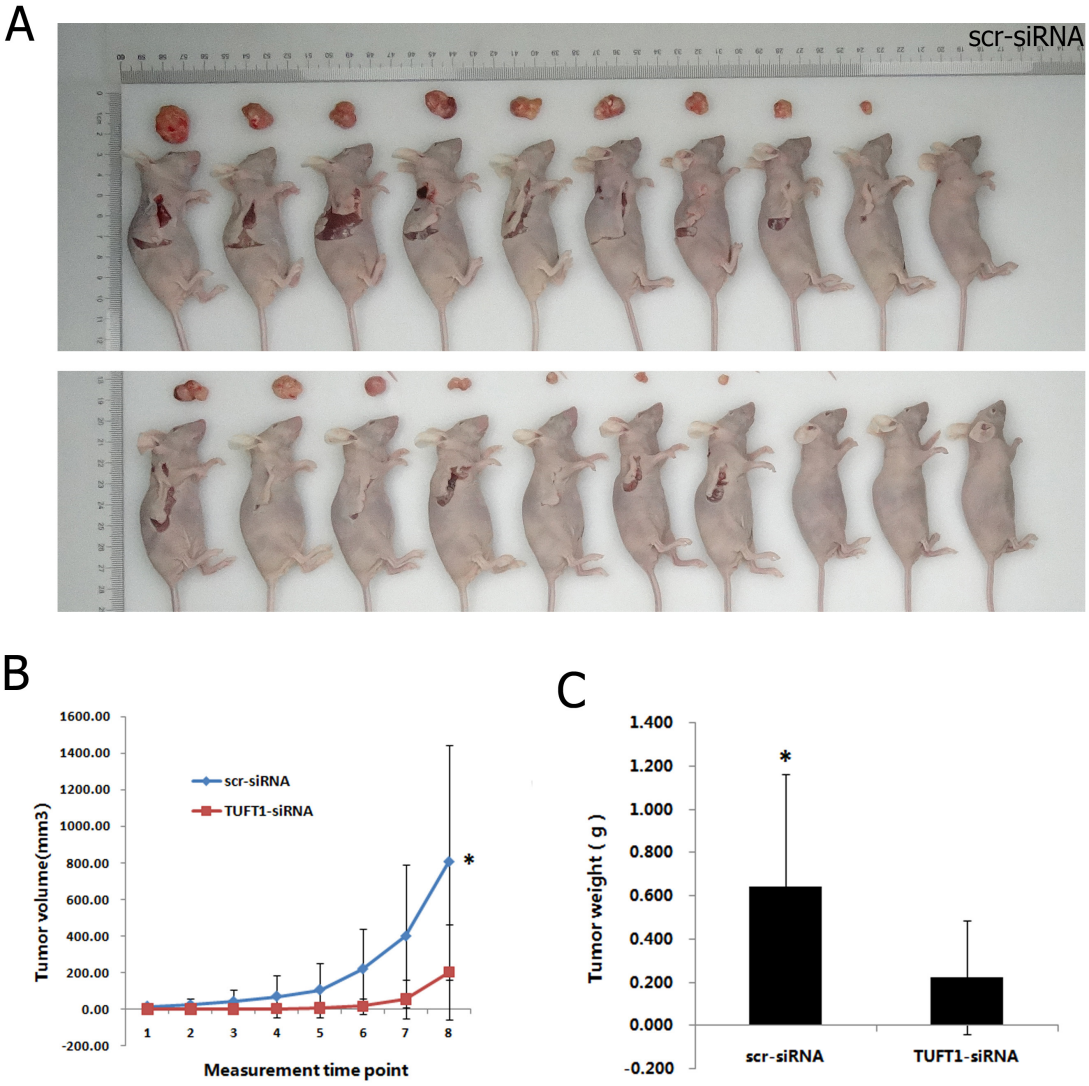
Effects of TUFT1 knockdown on xenograf tumorigenicity *in vivo* in nude mice Scr-siRNA and TUFT1-siRNA-expressing MDA-MB-231 cells were injected into the flanks of nude mice. Tumor growth **(A)** and tumor volume **(B)** were measured on the indicated days. Tumor weights were measured 10 weeks after injection **(C)**. Data shown are the mean ±S.D. (* t test P< 0.05, n = 10, respectively).

Scr-siRNA and TUFT1-siRNA-expressing MDA-MB-231 cells were implanted into nude mice, and tumor progression was monitored per week before the animals were sacrifced. The volume of TUFT1-siRNA MDA-MB-231 tumors was signifcantly smaller than control group throughout the experiment (P < 0.05) (Figure 7A, 7B). Similarly TUFT1-siRNA MDA-MB-231 tumors were lighter at the end of the observation period compared with control group (P < 0.05) (Figure 7C). These results suggest that TUFT1 plays a role in cancer growth *in vivo*.

### TUFT1 induces the activation of mitogen-activated protein kinase (MAPK) signaling

**Figure 8:**
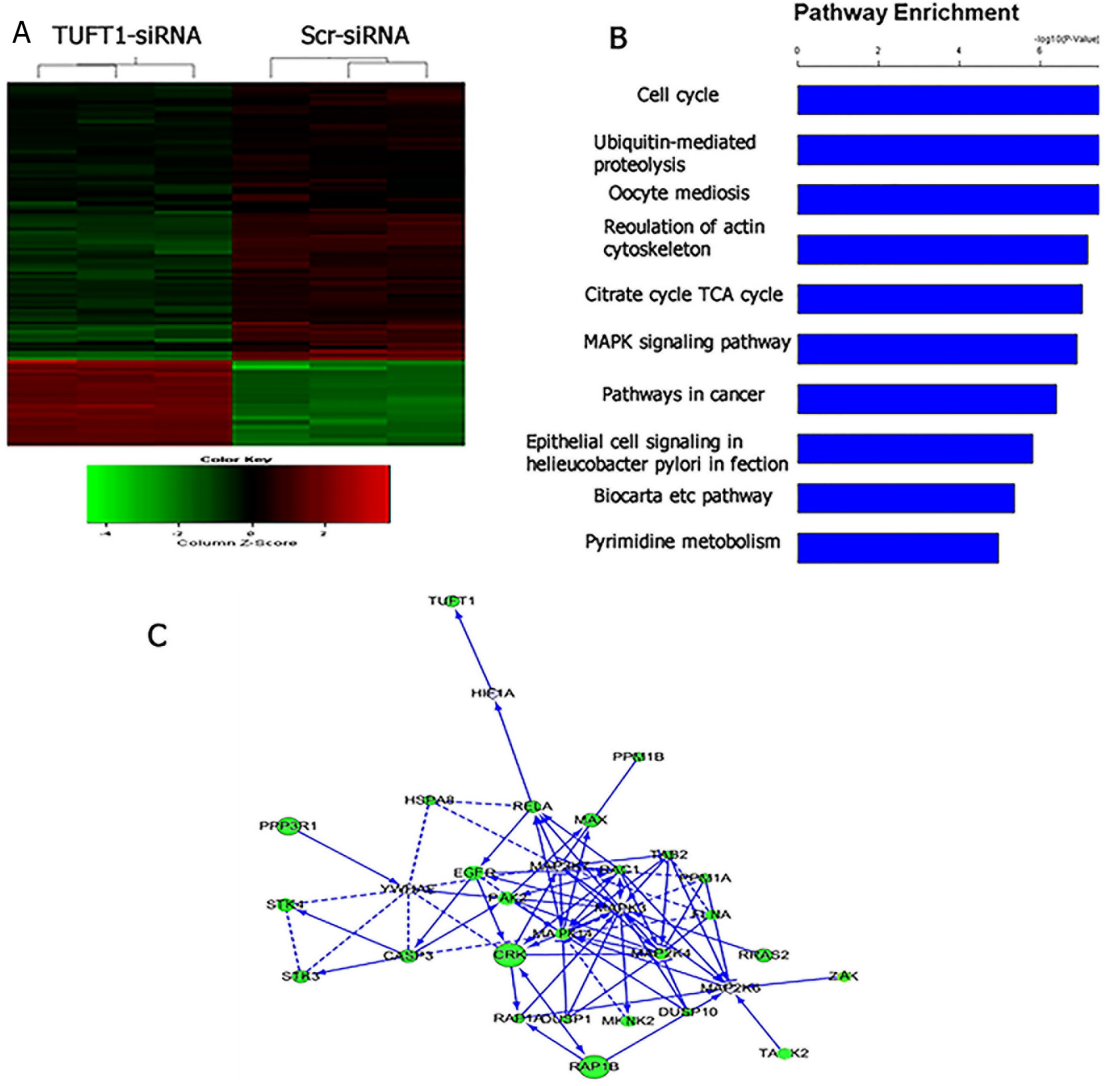
Widespread changes of gene expressions in MDA-MB-231 cells with TUFT1 knockdown by microarray **(A)** Heatmap depicting 1,095 genes that were significantly differentially expressed between cells transfected with scrambledand TUFT1-siRNA based on a P < 0.05. Rows and columns represent transcripts and samples, respectively. Upregulated anddownregulated gene expression is indicated by red and green colors, respectively. **(B)** Functional pathway analysis of the differentially expressed genes was conducted based on KEGG and BIOCARTA databases. Here, 10 significant-enriched pathways based on a P<0.001 were shown. The statistical significance shown in X axis is represented by the inverse log of the P value. **(C)** Networks were constructed between TUFT1 and genes involved in KEGG pathway MAPK. Solid arrow in the figure means confirmed regulatory relationships and dotted line means predicted regulatory relationships.

**Figure 9:**
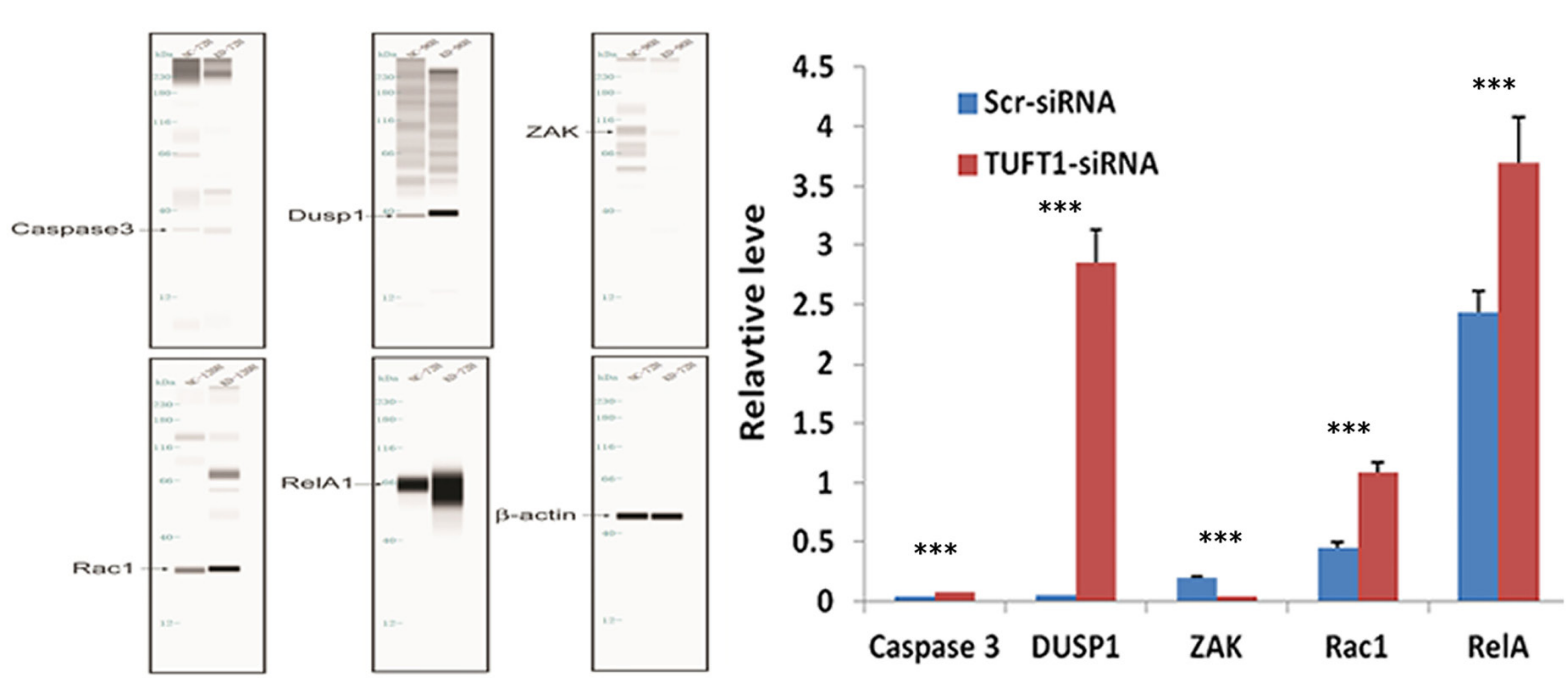
Confirmation of Caspase 3, DUSP1, ZAK, RAC1, RelA by automated western blot analysis system in MDA-MB-231 cells transfected with Scr- and TUFT1-siRNA Expression changes are depicted relative to cells transfected with Scr-siRNA. Data shown are the mean ±S.D. (***t test P <0.001 as compared to control groups, n = 3, respectively).

Furthermore, we compared the transcriptome of cells treated with TUFT1-siRNA with that in the control. Using a threshold of P < 0.05, we identified 1,095 genes that were shown to be significantly differentially expressed in TUFT1-knockdown MDA-MB-231 cells, and these included 278 genes shown to be upregulated and 817 downregulated genes (Figure 8A). Moreover, using the threshold of P < 0.001, we performed KEGG pathway analysis, which showed that these differentially expressed genes were significantly enriched in the 10 pathways. We selected MAPK signaling pathway (P = 1.24E-07; Figure 8B) for further analysis. Additionally, a significant induction of the expression of genes involved in MAPK signaling (*e.g*., RelA, caspase 3, DUSP1, RAC1 and ZAK) was observed, and functional interaction network analysis was further employed to investigate the relationship between TUFT1and genes involved in MAPK signaling( Figure 8C). After the selection performed by combining statistical and pathway analyses, their expression was confirmed by immunoblotting. The results showed that the expression of RelA, caspase 3, DUSP1, RAC1 was increased and ZAK was decreased in cells expressing TUFT1 siRNA (P<0.001, respectively, Figure 9).

## DISCUSSION

This is the first investigation into the role and mechanisms of TUFT1 in breast cancer, as far as we know. Bin Zhou [7] showed that TUFT1 is overexpressed in pancreas cancer (PC) tissues compared with adjacent normal pancreas tissues, and TUFT1 expression is significantly associated with lymph node metastasis and advanced PC stage. According to our results, TUFT1 protein levels were elevated in breast cancer tissues compared with adjacent normal tissues. In addition, we found that the positive TUFT1 expression was 16.7% in adjacent normal breast tissues, this revealed that TUFT1 could also express in some normal breast tissues. However, our IHC results showed that the positive staining intensity of adjacent normal breast tissues was mostly low, and the positive staining intensity of the tumor tissues was mostly medium or strong. These results indicate that TUFT1 may represent a potent marker for the improved breast cancer diagnosis. Generally, tumor size, histological grade, and the rate of lymph node metastasis are the evaluation of cancer cell proliferation and breast cancer prognosis [8, 9]. Here we showed that TUFT1 overexpression is positively correlated with tumor size, histological grade, and the rate of lymph node metastasis. Although the follow-up period was relatively short, TUFT1 overexpression was negatively correlated with the patient survival rate, indicating that TUFT1 may be involved in breast cancer progression, and serve as a prognostic factor for this type of cancer.

Afterward, we explored the effects and potential mechanisms underlying the activity of TUFT1 in breast cancer cells *in vitro* and *in vivo*. Our results indicated that the downregulation of TUFT1 expression can significantly affect cell proliferation and induce cell apoptosis. This is in accordance with the previously obtained results showing that TUFT1 is overexpressed in breast cancer samples. Additionally, we showed that the downregulation of TUFT1 expression may induce G0/G1 phase arrest in the investigated cells, regulating cell growth and blocking cell progression through the cell cycle. G1 phase is the key phase in which cell growth may be arrested or not [10–13]. Furthermore, cyclins A, B, D, and E interact with several cyclin-dependent kinases (CDKs), which are activated sequentially during the cell cycle. Cyclin D1 plays an important role in the early stage of the G1 phase of the cell cycle, and can regulate CDK4 and CDK6 [14, 15]. G1 phase arrest induced by TUFT1 downregulation may be related to the regulation of cyclin D1, CDK4, and CDK6. Furthermore, irreversible G1 arrest is the main marker of cell senescence. Several pathways, such as p16/pRb, p27, and p53/p21, are involved in the induction of senescence [13], and we postulate that TUFT1 knockdown-induced G1 phase arrest may be at least partially involved in the induction of cell senescence. However, the mechanisms underlying cell cycle arrest and apoptosis induced by TUFT1 downregulation in breast cancer cells remain unclear, and they should be further investigated.

In order to further study the mechanisms underlying TUFT1-induced effects in breast cancer, we used microarray analysis of TUFT1-knockdown MDA-MB-231 cells, and found significant differences in the expression of hundreds of genes, in comparison with that in the control cells. Gene chip and functional pathway analyses showed that TUFT1 expression is related to a variety of typical cancer-related pathways in human breast cancer cells. Moreover, we showed that ZAK expression was downregulated, while the expression of RelA, caspase 3, DUSP1, and RAC1 was upregulated following the siRNA-mediated TUFT1 knockdown in MAPK signaling pathway. The role of these genes has been reported in cancers [16–21], however, networks constructed between TUFT1 and genes had shown that TUFT1 did not associate with these genes except RelA. One previous study reported that RelA may regulate TUFT1 promoter in H9c2 cells, but the underlying mechanism is unclear [22]. Another study had found that TUFT1 may regulate epithelial mesenchymal transition by affecting HIF1-Snail signaling pathway in pancreas cancer [7]. So, more studies will be developed to explore the relationship between TUFT1 and these genes in breast cancer.

In conclusion, this is the first study linking TUFT1 expression and activity to breast cancer prognosis, and a positive correlation between TUFT1 expression and breast cancer cell proliferation and apoptosis was established for the first time here. Additionally, we showed that the alterations of the regulatory mechanisms affecting the expression of many oncogenes and the activation of cancer-related pathways may represent the mechanisms underlying TUFT1-dependent development and progression of breast cancer. TUFT1 may represent a novel molecular marker and potent therapeutic target for breast cancer treatment.

## MATERIALS AND METHODS

### Human specimens and cell lines

Here, we enrolled 146 patients with invasive breast cancer, undergoing breast surgery at the First Affiliated Hospital of the China Medical University, between January 2010 and December 2010. The criteria used for collecting patient samples were as follows: (1) radical surgery and postoperative adjuvant therapy were applied; (2) all samples underwent detailed pathological examination; (3) more than 9 lymph nodes were pathologically examined; and (4) all patients included in our groups were followed for the complete duration of the pre-determined follow-up period. The mean age of the patients was 50.37 years (range, 26-77 years). Our study and all experimental methods were approved by the Ethics Committee of China Medical University.

Human breast cancer cell line MCF-7, T-47D, MDA-MB-231, were obtained from Chinese Academy of Sciences (China), American Type Culture Collection (USA), American Type Culture Collection (USA), respectively. Three cancer cells were cultured in RPMI-1640 with 10% fetal calf serum (FCS), and incubated in a 37°C in an atmosphere containing 5% CO_2_.

### IHC analyses

We purchased rabbit polyclonal anti-human TUFT1 antibody from RayBiotech. All breast cancer samples were dewaxed using xylene, dehydrated using an alcohol gradient, and cleaned with phosphate-buffered saline (PBS). After antigen retrieval, the slices were incubated overnight at 4°C with the primary antibody. After an additional washing with PBS, the secondary antibody was added (poly peroxidase-anti-mouse/rabbit immunoglobulin; Maixin Biotech Co., Ltd.), and the samples were incubated at room temperature for 30 min. The results were obtained by using 3,3-diaminobenzidine catalysis. Slices were loaded, stained with Gill’s hematoxylin, and fixed in ethanol and xylene. In negative control, primary antibody was replaced with PBS. We considered TUFT1 immunoreactivity as positive when the cancer cytomembrane and cytoplasm were stained homogenously. We used a dual scoring system of staining extent and intensity for immunohistochemical analysis.

According to the scores of colored TUFT1 positive staining intensity: 0, no staining; 1 points, light yellow, slightly higher than the background color; 2 points, deep yellow; 3 points, dark brown.

According to the percentage of positive cells of TUFT1 staining: 0, no positive cells; 1 points, positive cells less than 25%; 2 points, 26-50% positive cells; 3 points, 51-75% positive cells; 4 points, more than 75% positive cells.

Multiplication of results of the above two methods is the final score: 0-2 points, negative (-); 3-4 points, weak positive (+); 5-8 points, moderately positive (+ +); 9-12 points, strong positive (+ + +). Two pathologists, working independently, performed these analyses.

### TUFT1 knockdown in T-47D and MDA-MB-231 cells

siRNAs, which can specifically target TUFT1, were designed, and lentiviral particles carrying TUFT1-siRNA were prepared (GeneChem, Shanghai, China). TUFT1 target sequence used here was: AGA GAA TTT AGA GAT GCA T, and scr-siRNA, used as a negative control, target sequence was: TTC TCC GAA CGT GTC ACG T. Initially, single-stranded oligoDNA sequence was synthesized and annealed in order to produce double-stranded oligoDNA, which was then directly linked to the RNA interference lentiviral vector. We used Lentivector Expression Systems (GeneChem, Shanghai, China) to prepare lentiviral particles carrying TUFT1-siRNA or scr-siRNA. Infection efficacy was determined by following the GFP expression under fluorescence microscope. Cell lines with over 80% efficacy were considered stable.

### RNA extraction and quantitative real-time PCR

Total RNA was extracted with Trizol (Invitrogen), which was followed by reverse transcription, according to the manufacturer’s instructions (Invitrogen). Quantitative real-time PCR was used to determine TUFT1 expression levels using the following primers: TUFT1, forward: TCA GAC TGT GTG GCT TTT GAG, reverse: GTC AGC ATT GTT GCT CCG AAG; GAPDH, used as a control, forward: TGA CTT CAA CAG CGA CAC CCA, reverse: CAC CCT GTT GCT GTA GCC AAA.

### Western blot

Cells were collected following the trypsin digestion, washed with PBS, and lysed with radioimmunoprecipitation assay (RIPA) lysis buffer with phenylmethane sulfonyl fluoride (PMSF). Total protein levels were determined by using BCA Protein Assay Kit (Pierce). The same amount of protein was taken from each sample, the same volume of 2× loading buffer was added, and the sample was cooked in boiling water for 10 min. Proteins were separated on 10% SDS-PAGE, and transferred to polyvinylidene fluoride (PVDF) membranes (Amersham), which were blocked with Tris-buffered saline with Tween 20 (TBST) solution containing 5% skimmed milk for 1 h, and the membranes were incubated with primary antibodies overnight. PVDF membranes were incubated with the secondary antibodies for 2 h, and the results were visualized using the ECL Plus Western Blotting kit (Amersham).

In the signaling pathway analyses, proteins were analyzed by using an automated western blot system (http://www.proteinsimple.com/simon.html). The following automatic ran was used: separate/immobilize/incubate with primary antibody/wash/ incubate with secondary antibody/wash/ incubate with enzyme substrate/expose [23–25].

### Analysis of cell proliferation

After infecting the cells with lentiviral vectors carrying TUFT1-siRNA or scr-siRNA, cells collected by trypsinization during the logarithmic growth phase and resuspended in complete medium. Afterward, cells were plated in 96-well plates (2000 cells/well), and incubated at 37°C. At day 2, Cellomics ArrayScan VTi (ThermoFisher) was applied, and cell growth was monitored continuously for 5 days. The obtained data were plotted, and three replicate wells were used for each group.

For MMT assay, 1.5 × 10^4^ cells/mL were plated in 96-well plates, and their proliferation was observed for 5 days. From day 2 after plating, to determine the cell proliferation rate at each time point, 10 μL MTT (5 mg/mL) was added per well, and the samples were incubated for 4 h. Following this, 100 μL of DMSO was added per well, and results were measured using a microplate reader, at 490 nm. Three replicate wells were used for each group.

### Cell cycle analysis

T-47D and MDA-MB-231 cells were collected upon reaching approximately 85% of confluence, in 5-mL tubes, and they were fixed for at least 1 h in 70% alcohol at 4°C. Cells were stained using 1∼1.5 mL of the following mixture: 40 × propidium iodide (PI)solution (2 mg/mL): 100 × RNase solution (10 mg/mL): 1× PBS = 25:10:1000. Flow cytometry (FACS Calibur, BD Biosciences, USA) was used for cell cycle analysis, and we aimed to achieve the cell pass rate of 200∼350 cells/s. Three replicate wells were used for each group.

### Apoptosis assay

T-47D and MDA-MB-231 cells were collected in 5-mL centrifuge tubes, after trypsinization, when the cells reached about 85% of confluence. Cells were washed with PBS, and cell pellet was resuspended in 1 mL of 1× staining buffer (final density of 1 × 10^6^ – 1 × 10^7^ cells/mL). Annexin V-APC (5 μL) was added to 100 μL of cell suspension at room temperature, and the samples were incubated in dark for 15 min. Flow cytometry (FACS Calibur) was used for further analysis, and three replicate wells were used for each group.

### Tumour growth in nude mice

MDA-MB-231 cells (1× 10^7^) were trypsinized and washed into single cell suspension with serum-free medium, and implanted subcutaneously into the flanks of BALB/c female nude mice (10 mice/group, 4 weeks old; Shanghai Lingchang Biological Technology Ltd, Shanghai, China). All mice were monitored once every 7 days and were sacrifced afer 10 weeks. All procedures were approved by the animal care and use committee of the First Hospital of China Medical University, and all experiments were performed in accordance with the approved guidelines.

### Path array analysis

Total RNA was extracted from MDA-MB-231 cells infected with lentiviral vectors carrying either scr-siRNA or TUFT1-siRNA, using Trizol reagent. RNA was evaluated using NanoDrop 2000 and Bioanalyzer Agilent 2100. The samples were selected for further experiments. Both cDNA strands were synthesized by reverse transcription. Afterward, labeled cRNA was synthesized by *in vitro* transcription, using GeneChip 3′IVT Expression Kit. GeneChip Hybridization Wash and Stain Kit were used for hybridization, washing, and staining. GeneChip Scanner 3000 was used to scan the arrays, in order to perform data evaluation. Gene expression profiling was performed using sing Affymetrix Human Gene 1.0 ST platform. The criteria used to determine the genes expressed differentially between MDA-MB-231 cells infected with TUFT1-siRNAs or scr-siRNAs, were P < 0.05 and |FC| > 1.5. KEGG and BIOCARTA data were used to perform pathway enrichment analysis.

### Statistical analysis

The correlation between TUFT1 expression and patient survival was analyzed using the Kaplan-Meier method. RT-PCR, western blot, MTT, cell cycle, and apoptosis assay results were analyzed using the Student’s*t*-test. χ^2^ analysis was used to assess the correlation between TUFT1 expression and tumor size, histological grade, and the rate of lymph node metastasis. P value of < 0.05 indicates a significant difference.
